# Addressing Heart Disease and Stroke Prevention Through Comprehensive Population-Level Approaches

**Published:** 2008-03-15

**Authors:** J Nell Brownstein

**Affiliations:** Division for Heart Disease and Stroke Prevention, Centers for Disease Control and Prevention

More than 79 million Americans have cardiovascular disease (CVD); it is the leading cause of death in the United States (1). In 2004, CVD was responsible for almost 40% of all deaths among Americans; 15.8 million adults had heart disease, 7.9 million had heart attacks, and 5.7 million had strokes (1). Although the complex constellation of risk factors, conditions, and diseases that constitute CVD is not easily understood by the public, most people are familiar with the terms *heart attack* and *stroke*.

The Division for Heart Disease and Stroke Prevention (DHDSP) of the Centers for Disease Control and Prevention (CDC) provides public health leadership in promoting cardiovascular health, reducing the burden of CVD, and eliminating disparities associated with heart disease and stroke (1). DHDSP's implementation of the recommendations in *A Public Health Action Plan to Prevent Heart Disease and Stroke* (2) promotes and helps achieve national prevention goals for heart disease and stroke through partnerships with public and private health agencies and others, in part through the National Forum for Heart Disease and Stroke Prevention (2). DHDSP and state, federal, and nongovernment partners conduct research and surveillance of CVD morbidity, mortality, and quality of care through, for example, the Paul Coverdell National Acute Stroke Registry in 33 states (2-4). DHDSP provides training, technical assistance, and funding to increase and improve the capacity of state health departments to combat these killer diseases. DHDSP funds the District of Columbia and 33 states in the National Heart Disease and Stroke Prevention Program and 14 states in the Well-Integrated Screening and Evaluation for Women Across the Nation (WISEWOMAN) Program (4).

DHDSP addresses heart disease and stroke across the prevention continuum through multipronged approaches and across multiple levels of influence on population health (2,5-13). In addition, DHDSP collaborates with national and state partners to affect systems-level policies on strategies that potentially can change behaviors of substantial numbers of Americans where they live, work, play, learn, and obtain health care (2,5,13). The theme articles in this issue of *Preventing Chronic Disease* reflect the focus of DHDSP and its partners on prevention, detection, and treatment of risk factors and conditions contributing to heart disease and stroke (e.g., high blood pressure and high blood cholesterol); early identification of stroke and heart attack; systems of care that directly impact heart disease and stroke; and cross-cutting tools and resources for program planning, implementation, monitoring, and evaluation (14-25). Elimination of disparities is crucial to all these areas — thus its inclusion as an overarching goal for *Healthy People 2010*.

## Prevention, Detection, and Treatment of Risk Factors for CVD

Reaching populations that are disproportionately affected by CVD and its risk factors is an ongoing challenge in the United States. Two articles highlight partnerships with organizations and communities for preventing, detecting, and treating risk factors. Wallace and colleagues describe the strong community partnership process used by the National Heart, Lung, and Blood Institute to produce culturally tailored and reader-friendly curricula that meet the special needs of disparate populations (14). Will and Loo note a significant challenge for WISEWOMAN: how to implement evidence-based interventions with maximum effectiveness for low-income women in diverse populations and communities (15).

Evidence is increasing that comprehensive programs offering risk-reduction counseling and using low-cost policy and environmental interventions effectively support healthy lifestyles and prevent CVD (26-28). One paper focuses on the importance of a comprehensive approach: Brissette and colleagues report on the New York program's statewide survey of 832 worksites with comprehensive wellness programs that include on-site services such as health risk appraisals; preventive blood pressure and cholesterol screenings; and support for healthy eating, physical activity, and stress management (16).

## Identification of Heart Disease and Stroke

Several articles highlight approaches for the early identification of heart attack and stroke. The results reveal that much work remains, especially in improving population-based knowledge about the warning signs of heart attack and stroke, the need to call 9-1-1, and the understanding that emergency medical services (EMS) are the optimal means of accessing urgent medical care. Like Maine (20), New York (17) found that public knowledge about stroke symptoms is low. New York's survey results show that delay in calling 9-1-1 is one of the most important obstacles to positive stroke outcome. Reasons for delay are similar to reasons for delayed response to heart attacks (29). Other findings from New York indicate the need to educate patients during routine medical visits and other teachable moments about the warning signs of stroke, the need for immediate medical attention, and the need to call EMS. Authors with the Massachusetts Heart Disease and Stroke Prevention Program describe the Stroke Heroes Act FAST media and public awareness campaign, which shows promise for stroke and 9-1-1 awareness (18). Such efforts are important because no attempts to improve EMS and hospital emergency department systems of stroke care will improve patient outcomes unless people recognize the warning signs of stroke and heart attack and activate the system by calling 9-1-1.

## Systems of Care for Heart Disease and Stroke

The Institute of Medicine (IOM) identified the fragmented U.S. emergency care system and called for creation of coordinated, regionalized, and accountable emergency care systems that include "protocols for the treatment, triage, and transport of prehospital patients" (30). Several articles in this issue of *Preventing Chronic Disease* address the need for improved systems of care that directly impact heart disease and stroke. Results from two Minnesota stroke care surveys show the state's progress in EMS recognizing stroke as an emergency event (19) — but the surveys also raise concerns about the differences between small rural hospitals and large metropolitan hospitals in terms of practices and capacity for stroke care (EMS and hospital).

Tracking patient outcomes and hospital services from 9-1-1 calls to hospital discharge is a major step in building collaboration between EMS and hospitals. Use of these data can strongly impact patient survival. Maine reveals a proactive cardiovascular health program that it conducts in collaboration with its EMS providers to share data to assess EMS outcomes (20). The links across systems of stroke care provide important information for public health officials on how people in communities access health care, and the problems they have using health care, as well as disparities between communities in how people seek and receive emergency care.

IOM has recommended establishing coordinated systems of care that integrate prevention and treatment services using evidence-based care (31). The American Stroke Association calls for providers and policymakers "to promote coordinated systems that improve patient care" (32). George and colleagues demonstrate the key role of state health departments in assessing and ensuring quality of care for acute stroke (21). State health departments can further develop systems of stroke care through quality improvement initiatives and partnerships to coordinate statewide stroke policies.

## Tools

The final set of articles presents tools, resources, and approaches to assist program planning, priority setting, monitoring, and evaluation. Understanding national policies that might help reduce the burden of CVD is a challenge. Ford Lattimore and colleagues highlight a new tool that provides state CVD and stroke prevention and other programs with a policy database, a mapping application, and other tools to aid development, implementation, and evaluation of legislative policy (22).

Matson Koffman and colleagues explain the *Purchaser's Guide to Clinical Preventive Services*, an information source developed by CDC and the National Business Group on Health (23). The *Guide* synthesizes supporting evidence, strategies for prioritization, and recommendations by which, in this era of limited resources and increased accountability, employers can improve delivery and use of preventive services and design value-based quality health plans for their employees.

Homer and colleagues describe a system dynamics modeling approach for cardiovascular health (24). This method helps address the fundamental problem of knowing where to act in the complex CVD "system" for highest impact (33). DHDSP is exploring system dynamics modeling in Austin, Texas, by partnering with local leaders to test and compare potential interventions and to help provide the community with information about preventing heart disease and stroke.

Sitaker and colleagues describe the development of the Washington state heart disease and stroke program logic model and its use to generate the program evaluation plan (25). They demonstrate the utility of logic models in heart disease and stroke programs and discuss how logic models can provide information about the evolution of evaluation plans.

DHDSP is helping states build their capacity to collect and use data to prevent and manage CVD and to partner with stakeholders to build strong, integrated systems of evidence-based care for stroke, heart attack, and heart disease (18,21-23,26). States also are developing and testing public education campaigns, especially for populations at highest risk, to increase awareness of risk factors, warning signs of heart attack and stroke, and the need to call 9-1-1 for immediate medical attention (16). The states' data highlight the need for improvements in public and professional education, public policy, and coordinated systems of patient care; these data are critical for the states' program planning and priority setting, for identifying populations at highest risk for CVD, and for informing policy makers. Preventive and medical services for low-income women in the community (through WISEWOMAN) and social support and education (through the curricula of the National Heart, Lung, and Blood Institute) provided by trained lay persons to members of disparate communities illustrate attempts to influence communities through social environments (14,15). In addition, DHDSP provides states with necessary tools and resources (e.g., evaluation, policy database, employer guide to clinical preventive services) (27-31).

These actions by DHDSP and its state and national partners illustrate our attempts to collaboratively affect the complex confluences of population-level cardiovascular health. DHDSP continues to work to improve cardiovascular disease outcomes and to reduce death and disability from heart disease and stroke to attain the vision of a heart-healthy and stroke-free nation.
